# Effects of three orthodontic retainers on periodontal pathogens and periodontal parameters

**DOI:** 10.1038/s41598-023-46922-2

**Published:** 2023-11-24

**Authors:** Bowen Li, Cailian Lu, Xinhui Yao, Xiaojun Wu, Guilin Wu, Xiantao Zeng

**Affiliations:** 1 Institute of Oral Science, Department of Stomatology, Longgang Otorhinolaryngology Hospital, Shenzhen, 518172 People’s Republic of China; 2 Department of Stomatology, The First Hospital of Shanxi Medical University, Taiyuan, 030001 People’s Republic of China

**Keywords:** Diseases, Health care, Dentistry

## Abstract

The objective of this study was to compare and evaluate the changes in periodontal pathogens and periodontal status within 6 months of wearing three orthodontic retainers, namely, vacuum-formed retainer (VFR), Hawley retainer (HR), and lingual fixed retainer (LR). In total, 48 patients who underwent orthodontic treatment with ordinary metal brackets were divided into VFR, HR, and LR groups (n = 16 per group). Saliva samples were collected at the time of debonding (T0) and after 1 month (T1), 3 months (T2), and 6 months (T3). *Porphyromonas gingivalis* (Pg) and *Aggregatibacter actinomycetemcomitans* (Aa) were quantitatively analyzed using real-time PCR. Gingival index (GI), plaque index (PLI), and probing depth (PD) were measured at the four time points to evaluate changes in periodontal state. SPSS20.0 software was used to analyze the data, and *P* < 0.05 was considered statistically significant. The trial was registered at the Chinese Clinical Trial Registry (ChiCTR2300073704), the registration was retrospective. Compared to baseline (T0) values, Pg, Aa, GI, PLI, and PD were significantly decreased in all three groups 1 month after wearing the retainer (*p* < 0.05). Significant differences were observed in Aa at T3 among the three groups, whereby the HR group exhibited significantly better results compared to the VFR and LR groups (*p* < 0.05). Differences were found among the three groups’ *Porphyromonas gingivalis* at T3, and the HR group was significantly better than the VFR and LR groups (*P* < 0.05). From T1 to T2, GI, PLI, and PD of the three groups tended to be stable, however differences were observed at T3, with the PLI and PD of the HR group being the lowest among the three groups (*p* < 0.05). Regardless of the type of retainer used, the periodontal condition of patients was significantly improved after removal of the metal brackets. After 6 months of retainer use, the Hawley retainer was superior to vacuum-formed retainer and lingual fixed retainer with regard to Pg, Aa, and periodontal clinical parameters.

## Introduction

After orthodontic treatment, periodontal tissue and alveolar bone remodeling occurs more slowly than changes in the teeth, and are affected by occlusal interferences and growth factors, the tooth will bounce back to its starting position^1^. Studies have shown that about 70 to 80 percent of orthodontic patients have a relapse for a variety of reasons^2^. Therefore, the use of orthodontic retainers is a critical component of orthodontic treatment and various orthodontic retainers are currently available for clinical application. However, the retainer is a foreign body in the oral environment, which may disturb the dynamic balance of oral microbes, and consequently affect periodontal health.

In the process of orthodontic treatment, the use of fixed orthodontic appliances may hinder cleaning measures and self-cleaning oral hygiene practice, resulting in increased risk of plaques, changes in oral microflora, deterioration of oral hygiene, and gingival inflammation^3, 4^. In the maintenance phase of orthodontic treatment, several issues remain of concern to both doctors and patients, including whether the use of various retainers can restore periodontal tissue health, the duration of restoration, the choice of retainer with the least impact on periodontal tissue, and the risk of damage to periodontal tissue. Currently, vacuum-formed retainers (VFR), Hawley retainers (HR), and lingual fixed retainers (LR) are commonly used in clinical practice.

Studies have reported that orthodontic treatment may cause an imbalance in the local oral microecology, resulting in changes in the oral flora of patients^5, 6^. A proportion of conditioned pathogenic bacteria may become dominant and contribute to periodontal tissue changes, such as gingivitis and periodontitis. The occurrence and development of periodontitis are related to various subgingival pathogenic bacteria, such as *Porphyromonas gingivalis* (Pg) and *Aggregatibacter actinomycetemcomitans* (Aa), which are well-documented periodontal pathogens^7^. Pg is one of the most widely studied and well-documented periodontitis—causing bacteria. Pg has the highest detection rate of subgingival plaques in patients with chronic periodontitis, and has also been detected in healthy individuals^8^. Aa is associated with adolescent periodontitis as well as other types of periodontitis, and can also be detected in healthy individuals. Growing evidence suggests that Aa is closely associated with invasive periodontitis^9^.

Developments in biotechnology have improved methods for detection of periodontal microorganisms. In particular, real-time PCR, a relatively novel method based on conventional PCR detection, is typically employed to determine the number, and species, of specific bacteria^10^. The basic principle of this approach involves the addition of fluorescent substances that specifically mark PCR products into the PCR reaction, and harnessing the accumulation of fluorescence signals to monitor the entire PCR process in real time. This process produces an S-type amplification curve. If the initial PCR curve conforms to exponential amplification, the molecular number of the initial template can be determined indirectly by comparing the cumulative time of products based on a simple exponential equation, and the template can be quantitatively analyzed using a standard curve^11, 12^.

Recent studies have reported retention effect, survival, and patient satisfaction with different retainers^6, 13, 14^. However, there is a paucity of studies on periodontal tissue recovery after removal of fixed appliances, and quantitative changes in periodontal pathogens during appliance wear. Further, in a 2016 Cochrane review of orthodontic retainers, the authors emphasized the need for more studies to compare the effects of different retainers on periodontal conditions^15^.

## Materials and methods

### Study population

The trial was registered at the Chinese Clinical Trial Registry (ChiCTR2300073704), but a retrospective registration was performed in July 2023. The study procedures were approved by the Medical Ethics Committee of the first Hospital of Shanxi Medical University in November 2019 (No.2019k033). All participants were informed about the experimental procedure and signed written informed consent form in Chinese. If the participant is a minor, consent must be obtained from the guardian. Participants were recruited to the study from November 2019 through November 2021. The study was conducted from October 2019 through May 2022.Because of the single-blind nature of the experiment, the investigators were able to identify information about the participants at return visits. All methods were performed in accordance with the relevant guidelines and regulations.

The study population consisted of healthy patients who had successfully completed orthodontic treatment with fixed orthodontics and were scheduled to enter the maintenance phase. The inclusion criteria were as follows: (1) both upper and lower dental arches were treated with fixed orthodontic treatment, and patients were satisfied with the orthodontic results, (2) patients used brackets with a 0.022-inch groove (3M Unitek) and common metal brackets, (3) more than 24 permanent teeth, (4) patients with good oral hygiene, and (5)orthodontic treatment > 12 months. The exclusion criteria were as follows: (1) systemic diseases, (2) use of any drugs that may have affected periodontal health (such as antibiotics, Chinese herbal medicine, and mouthwash can have direct or indirect effects on periodontal health), (3) severe or uncontrolled caries, (4) cleft palate or severe facial deformities, (5) any active periodontal disease, (6) dentures in the mouth, (7) smokers, and (8) pregnant patients.

The size of the population was predetermined by means of power analysis in G*Power version 4.1.1 software.On the basis of a 1:1 ratio between groups, the power of the three groups was more than 80% power (actual power 0.817) with a total sample size of 45, and the effect size was 0.40 at the significance level of α = 0.05.

**Figure 1:**
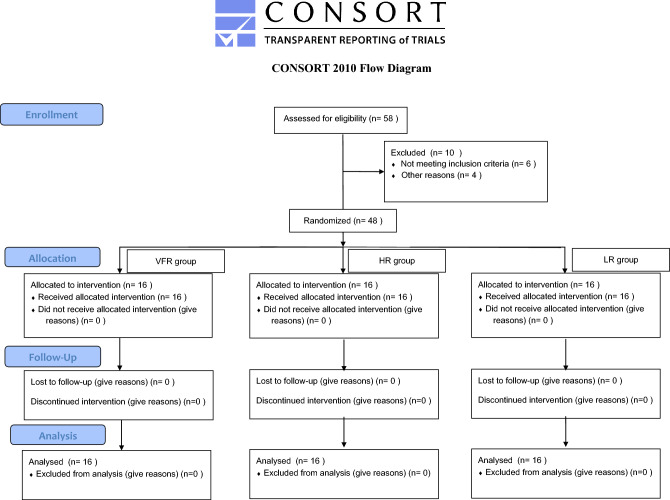
Consort 2010 flow diagram for patient selection. Ultimately, 16 patients in the VFR group, 16 patients in the LR, 16 patients in the HR group were analyzed.

Forty-eight patients who met the inclusion criteria and were about to complete their orthodontic treatment were randomly divided into 3 groups (Fig. 1). The order of enrollment of the study subjects was numbered. All samples were arranged in ascending order using “Microsoft Excel”. The "RAND" function was entered in the second column for randomization, which was divided into three groups: VFR, HR, and LR.

### Retainer types and evaluation parameters

**Figure 2:**
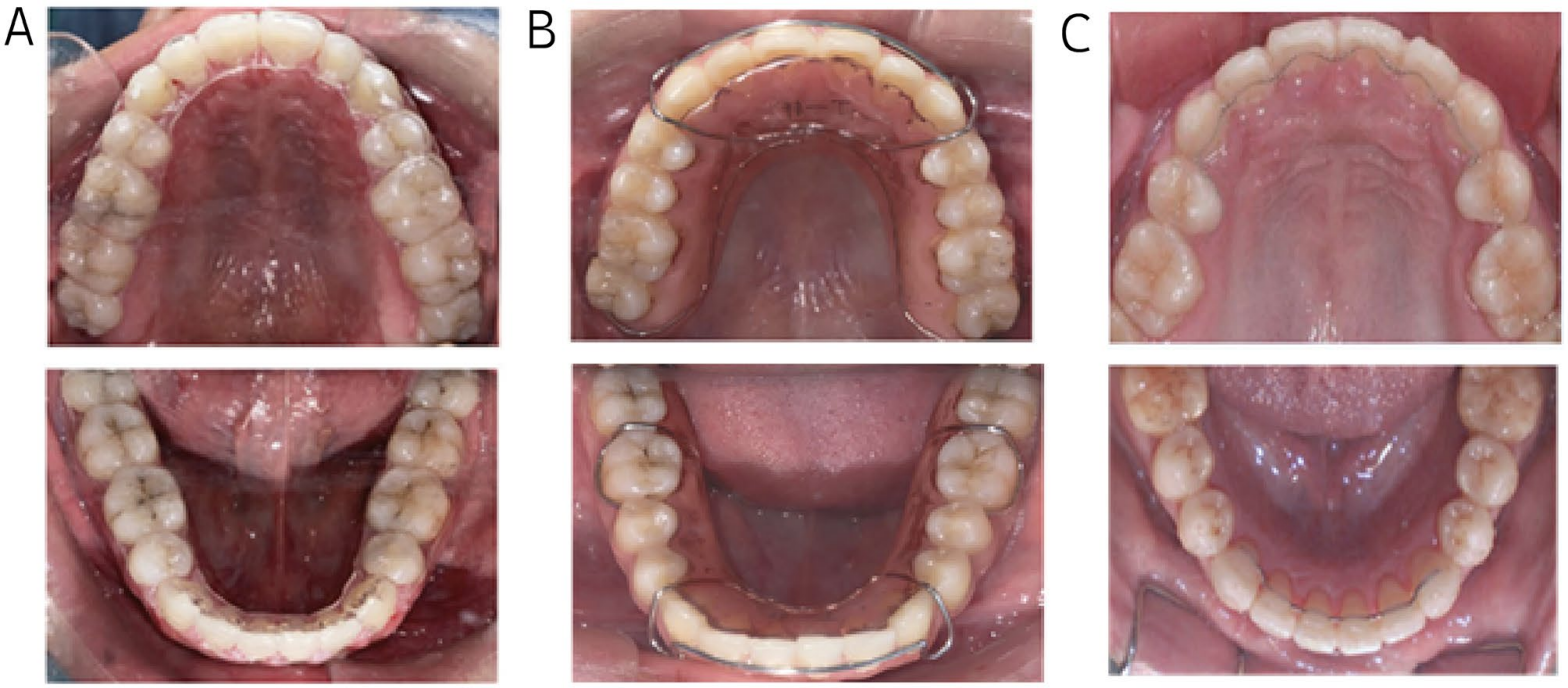
Three types of orthodontic retainers commonly used in clinical practice. (**A**) vacuum-formed retainer (VFR). (**B**) Hawley retainer (HR). (**C**) lingual fixed retainer (LR).

In the VFR (Splink; Shanghai, China) group, a 1 mm thick patch was used in the upper and lower jaw. The VFR was made of polyvinylchloride material produced using the thermoforming technique, covering all the surfaces of the teeth up to the gum margin. Patients were required to wear the VFR for more than 22 h a day, except for meals and tooth-brushing time. The HR was made of acrylic resin and a 0.8-mm stainless steel lip bow. Patients were required to wear the HR at all times, except when eating and brushing teeth. The LR (BetterHealth; Shanghai, China) was a flat twist of 0.0215-inch, three-strand nickel-titanium wire that was bonded to the lingual surface of the six anterior teeth using fluid resin (Songfeng; Shanghai, China). Care was taken to avoid contact of the twisting silk and adhesive with the gingival tissue. As the lingual fixator was bonded to the tooth, patients were required to wear it for 24 h a day (Fig. 2). Patients in the three groups were prescribed the same oral care methods, such as brushing their teeth in the morning and evening, using the Bass brushing method, gargling after eating, using a manual toothbrush to assist flossing and water flossing, and avoiding the use of mouthwash when wearing the retainer. Patients were required to strictly adhere to these methods.

Patients were assessed at removal of the bracket (T0) and after 1 month (T1), 3 months (T2), and 6 months of wearing the retainer (T3). Evaluation parameters included gingival index (GI), plaque index (PLI), probing depth (PD), and the periodontal pathogens content of Pg and Aa. All assessments and retainers were performed by two orthodontists trained as periodontists, they qualified as periodontists.

Index teeth 16, 21, 31, and 46 were selected to measure the periodontal clinical indicators. Patients were required to refrain from eating or brushing their teeth for 2 h before the test. In this study, the GI adopted the scoring standard proposed by Loe and Silness^16^, and the PLI adopted the scoring standard proposed by Quigley and Hein^17^. A detailed scoring standard is presented in S1 Appendix. PD was determined using the periodontal probe KPC15 (Kangqiao; Shanghai, China). The distance from the bottom of the periodontal pocket, or gingival trench, to the gingival margin was measured. Six sites were detected for each index tooth, with three measurements per site, and the average value was obtained. At the time of T0 sampling, the patient had not removed the metal bracket. The data collected at T0 therefore represented the patient's periodontal condition and bacterial status during orthodontics, and this timepoint was considered the baseline. Ultrasonic scaling was not performed after the brackets were removed.

### Sample preparation and real-time PCR

Saliva samples were collected by the same clinician at T0, T1, T2, and T3 prior to the assessment of periodontal parameters. Patients were required to refrain from eating, drinking, or brushing their teeth for 2 h before saliva collection. Approximately 2 mL of non-irritating saliva was collected in sterile test tubes and stored at − 80 °C for real-time quantitative PCR.

**Figure 3:**
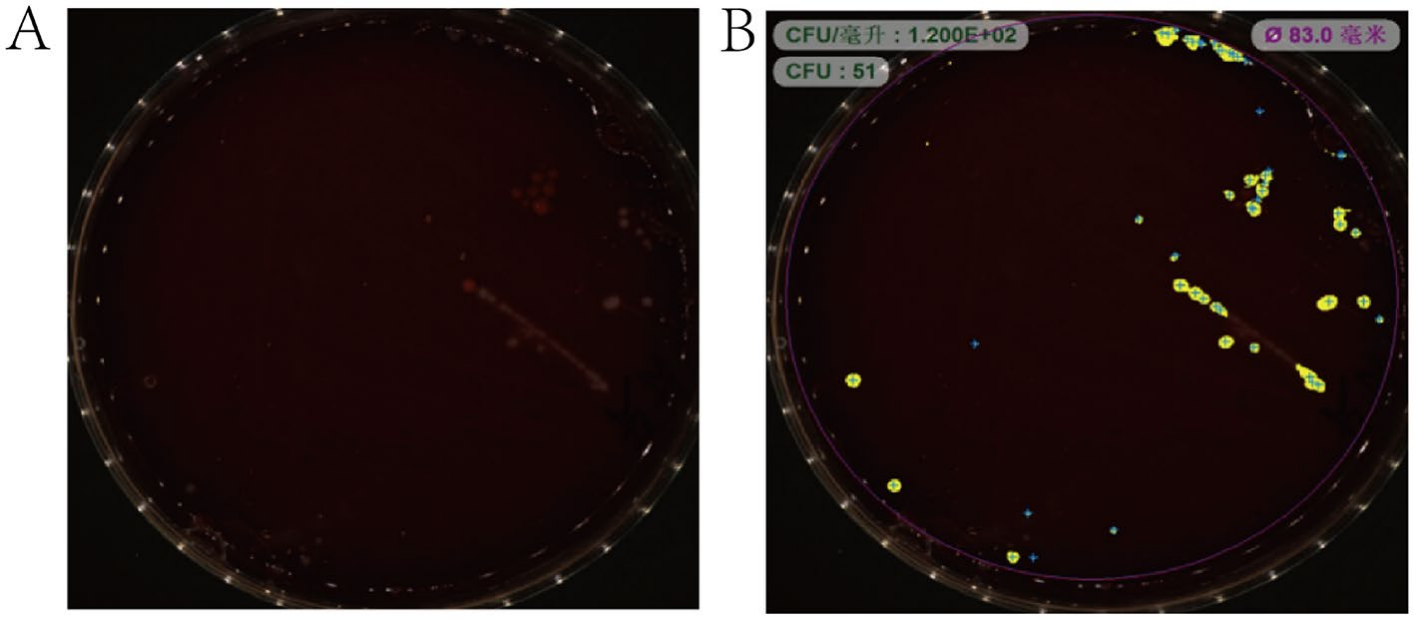
Standard strains were grown anaerobically. (**A**) Cultures were grown in Petri dishes. (**B**) The analysis was performed in an analyzer.

The standard strains used in this study were Pg ATCC 33,277 and Aa ATCC 43,718. Both strains were obtained from the Shanghai Conservation Biotechnology Center (SHBCC; Shanghai, China) and were cultured in Petri dishes (Nissui Seiyaku, Tokyo, Japan) (Fig. 3). Bacterial DNA was extracted from saliva samples using the Oral Swab Genomics DNA Extraction kit (Tiangen Biology; Beijing, China) according to the manufacturer’s instructions. Primers for Pg and Aa (Table 1) were detected and quantified using SYBR Green reagent. PCR primers for Pg and Aa were designed based on the 16S rRNA gene. All primers were artificially synthesized by Oligo USA, and sequence alignment was performed. Primer-specific BLAST homology analysis is presented in S2 Appendix and S3 Appendix. The DNA standard curves of Pg and Aa consisted of a known number of purified PCR products isolated from agar gels and diluted to a concentration of 1 × 10^3^ to 1 × 10^8^ copy number/μL. The amount of bacterial DNA in the samples was calculated using a standard curve and established quantitative criteria.

**Table 1:** Primer sequences used in real time PCR.

Gene	Primer	Sequence (5′-3′)	PCR products
P.g 16SrRNA	Forward	GACTAAAACCGCATACAC	198 bp
	Reverse	GACTTACCGAACAACCTA	
Aa 16SrRNA	Forward	GCGTAGAGATGTGGAGGAAT	430 bp
	Reverse	GGCAACAAAGGATAAGGGT	

Pg : P. gingivalis, Aa: A. actinomycetemcomitans.

**Figure 4:**
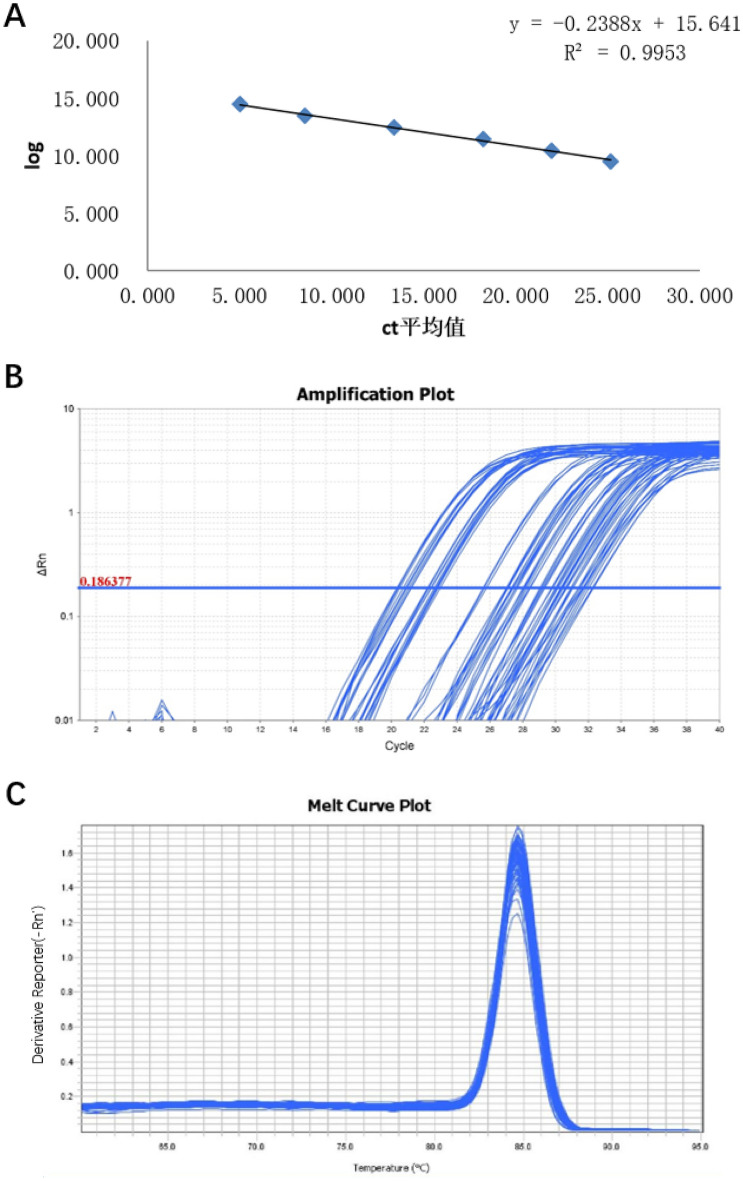
PCR curve of Pg. (**A**) The standard curve. (**B**) Amplification curve. (**C**) Melt curve.

**Figure 5:**
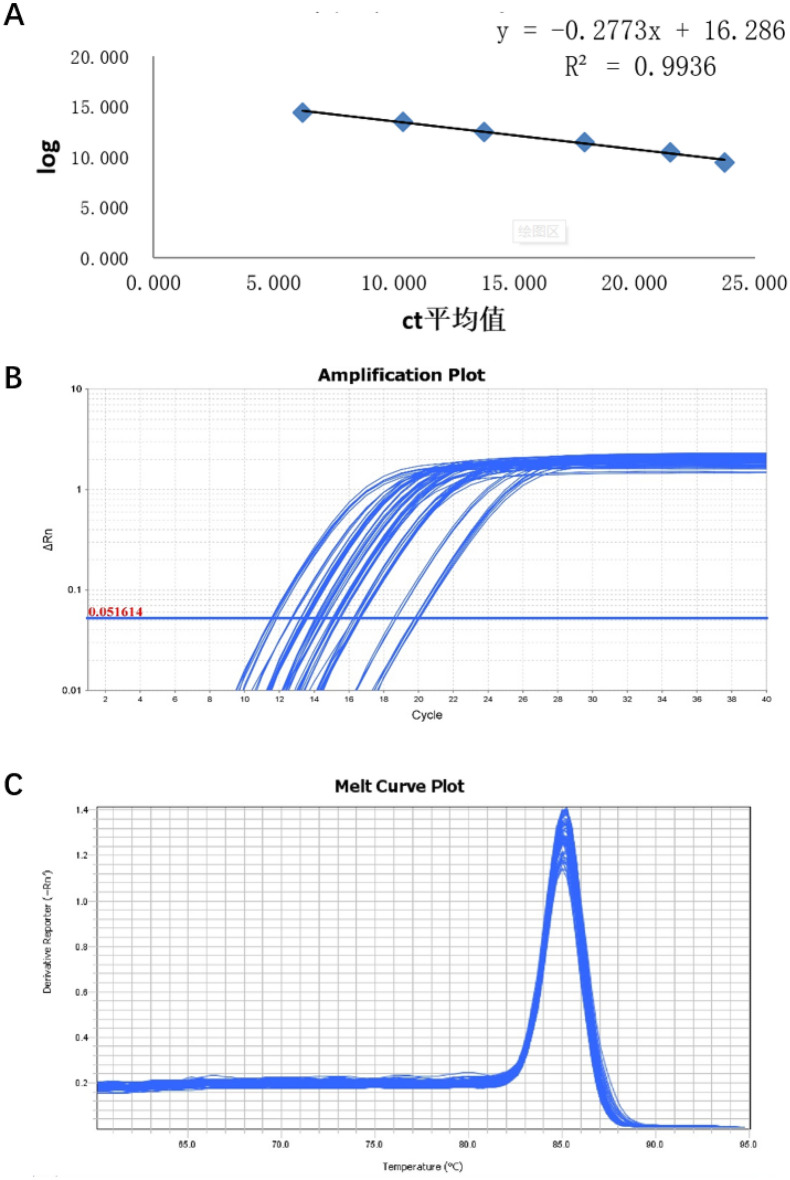
PCR curve of Aa. (**A**) The standard curve. (**B**) Amplification curve. (**C**) Melt curve.

In real-time PCR, Pg and Aa were detected and quantified using SYBR Green reagent. Test sample qRT-PCR assays were performed in a total volume of 20 μL. The reaction system and conditions are described in S4 Appendix. The reaction was performed using ABI PRISM7300 fluorescence quantitative PCR (Applied Biosystems, USA). Each sample was set in three wells. Ct values obtained from multiple wells were calculated using the ABI7300 SDS software, according to the standard curve, to obtain the initial DNA concentration. The values were subjected to logarithmic transformation, and the mean value was calculated. Blank control and standard positive controls were used for each reaction. The standard curve, amplification curve, and dissolution curve of Pg are shown in Fig. 4A–C. The standard curve, amplification curve and dissolution curve of Aa are shown in Fig. 5-A–C.

### Statistical analysis

The SPSS software package was used for data analysis (Version 20; SPSS, USA), with statistical significance set at 5%. Data were assessed for normality using the Shapiro–Wilk normality test. The Levene test was used to assess homogeneity of variance. The results of the normality test indicated that the data of periodontal parameters did not conform to a normal distribution, while the Pg and Aa data were normally distributed with a uniform variance. Therefore, the Kruskal–Wallis test was used for overall inter-group comparisons of periodontal parameters, whereas the Friedman test and Nemenyi post-test were used for intra-group comparisons, the data in the tables are described in quartiles. Univariate ANOVA was used for overall comparison of bacteria between groups, and Bonferroni-corrected test results were used for intra-group comparisons, the data in the table are described as mean ± standard deviation. Log 10 transformation was performed on the microbiologic data so that the distribution was normalized and the variance was stabilized. Spearman’s correlation coefficient was used to analyze the correlation between the periodontal clinical parameters, Pg, and Aa.

## Results

Between November 2019 and November 2021, a total of 48 patients participated in the study. The VFR, HR, and LR groups comprised 16, 16, and 16 patients, respectively. The mean ages of patients in the VFR, HR, and LR groups were 17.20 ± 1.56, 18.25 ± 1.36, and 17.45 ± 1.20 years, respectively, with no significant differences among the three groups (p > 0.05). The male: female ratios were 8: 8, 9: 7, and 9:7 in the VFR, HR, and LR groups, respectively, with no significant difference among the three groups (*p* > 0.05) (Table 2). All data generated or analysed during this study are included in this published article (and its supplementary files).

**Table 2:** Demographic Information on the Subjects in This Study.

Total patients (n)	48
Age	17.55 ± 1.42
Sex	26 M/22 F
Group A (n)	16
Age	17.20 ± 1.56
Sex	8 M/8 F
Group B (n)	16
Age	18.25 ± 1.36
Sex	9 M/7 F
Group C (n)	16
Age	17.45 ± 1.20
Sex	9 M/7 F

Data are means and standard deviations.

Group A, VFR.

Group B, HR.

Group C, LR.

M, Male; F, female.

**Figure 6:**
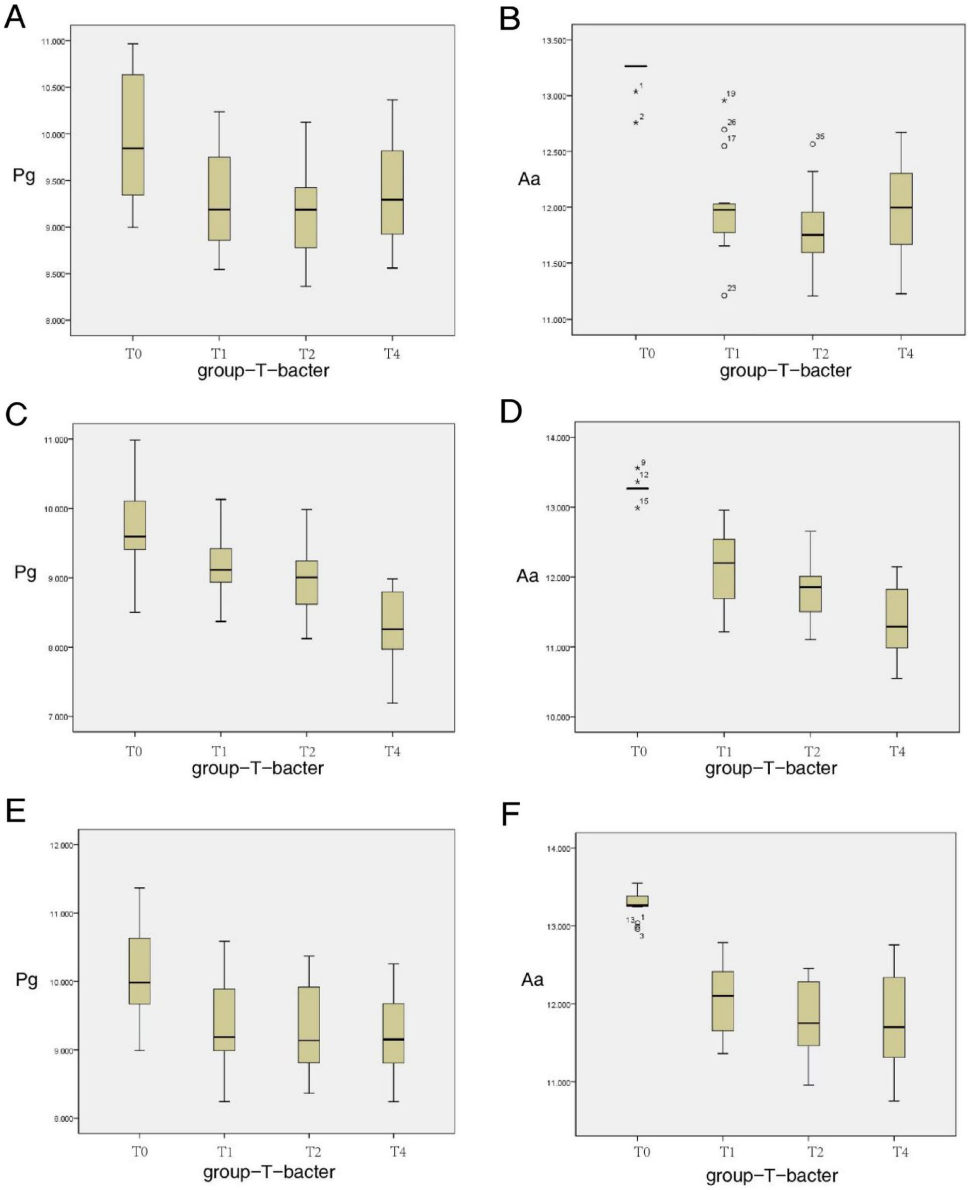
Box plots of bacterial changes in three orthodontic retainers at different times. (**A**) In the VFR group, the amount of Pg decreased significantly at T1 and then became stable. (**B**) In the VFR group, the amount of Aa decreased significantly at T1 and then became stable. (**C**) The change trend of Pg in HR group during the 6 months after bracket removal was significantly decreased at T1 and then decreased. (**D**) The change trend of Aa in HR group during the 6 months after bracket removal was significantly decreased at T1 and then decreased. (**E**) In the LR group, the amount of Pg decreased significantly at T1 and then became stable. (**F**) In the LR group, the amount of Aa decreased significantly at T1 and then became stable.

A statistical comparison of the changes in Pg and Aa counts in the three groups is presented in Table 3. At baseline T0, there were no significant differences in Pg and Aa among the three groups (*p* > 0.05). At 1 month after the removal of brackets, the Pg and Aa counts in all the three groups exhibited a significant decrease (T0 > T1, T2, T3, *p* < 0.05). Pg in HR group decreased gradually after T1, there were no significant differences at T1, T2, and T3 (*p* > 0.05). Pg in the LR and VFR groups remained stable at T1, T2, and T3 (both *p* > 0.05). There were no significant differences in Pg among the three groups at T1 and T2 (*p* > 0.05). However, Pg of HR group was lower than the LR and VFR groups at T3, and the difference was statistically significant (*P* < 0.05). In the HR group, Aa remained decreases after a decrease at T1, there were no significant differences at T1, T2, and T3 (*p* > 0.05), with the lowest value observed at T3. Aa in the LR group remained stable after a decrease at T1, there were no significant differences at T1, T2, and T3 (p > 0.05). Aa in the VFR group remained stable at T1 and T2 and at T3 (*p* > 0.05). There were no significant differences in Aa at T1 and T2 among the three groups (*p* > 0.05); however, at T3, Aa was significantly lower in the HR group than in the VFR and LR groups (*p* < 0.05). The bacterial changes of the three retainers were compared in different periods by box diagram (Fig. 6).

**Table 3:** Means, standard deviations, and statistical comparisons of the bacterial counts (log 10).

Bacteria	Group	n	T0		T1		T2		T3		*P* value*					
			Mean	SD	Mean	SD	Mean	SD	Mean	SD	T0–T1	T0–T2	T0–T3	T1–T2	T1–T3	T2–T3
Pg	VFR	16.00	9.96	0.65	9.31	0.52	9.19	0.54	9.31	0.54	0.01*	0.01*	0.01*	0.45	0.56	0.36
	HR	16.00	9.70	0.64	9.16	0.52	8.99	0.48	8.90	0.56	0.04*	0.02*	0.02*	0.47	0.54	0.38
	LR	16.00	10.15	0.72	9.37	0.65	9.29	0.66	9.24	0.63	0.01*	0.01*	0.02*	0.56	0.49	0.23
	*P* value		0.17		0.55		0.32		0.02#							
Aa	VFR	16.00	12.72	0.41	12.01	0.42	11.82	0.35	12.01	0.46	0.01*	0.01*	0.01*	0.32	0.45	0.62
	HR	16.00	12.81	0.39	12.11	0.49	11.79	0.43	11.36	0.50	0.02*	0.01*	0.01*	0.28	0.02*	0.07
	LR	16.00	13.18	0.49	12.04	0.47	11.80	0.47	11.82	0.59	0.01*	0.01*	0.01*	0.54	0.46	0.61
	*P* value		0.46		0.82		0.97		0.03#							

VFR, vacuum-formed retainer; HR, Hawley retainer; LR, Lingual retainer.

*Bonferroni multiple comparison test result (intragroup comparisons), Significant differenc.

^#^One-way analysis of variance (ANOVA) test result (intergroup comparison), Significant difference.

Table 4 presents the changes in periodontal parameters in the three groups. There were no significant differences in the periodontal clinical index values of the retainer between the three groups at baseline (T0). Compared to the baseline values at T0, PLI, GI, and PD values in all three groups at T1, T2, and T3 were significantly lower (T0 > T1, T2, T3, *p* < 0.05). In all three groups, PLI decreased in the first month after bracket removal. There was no significant difference in PLI at T1, T2 and T3 in Hawley group. PLI in LR group had no significant difference at T1, T2 and T3 (*P* > 0.05).However, PLI in the VFR group increased significantly at T3 (T3 > T1, T2, *P* < 0.05), but did not reach T0 baseline levels. At T3, the HR group had the lowest PLI, while the VFR group had the highest PLI, and the difference was statistically significant (*P* < 0.05). The GI of the HR and LR groups tended to be stable at T1, T2 and T3, and there was no significant difference between the two groups (*P* > 0.05). The GI of the VFR group was significantly increased at T3, but its value was lower than that of T0 (T0 > T3 > T1, T2, *P* < 0.05). There was no significant difference in GI between the three groups at T1, T2 and T3 (*P* > 0.05). The PD of the retainer decreased significantly in the first month after bracket removal in all three groups, and there was no significant difference in PD of the HR and LR groups at T1, T2, and T3 follow-up (*P* > 0.05); however, PD in the VFR group increased significantly at T3 (T3 > T1, T2, *P* < 0.05), but did not reach T0 baseline levels. At T3, the PD was compared between groups, and the HR group was the lowest, while the VFR group was the highest, and the difference between the groups was statistically significant (*P* < 0.05).

**Table 4:** Comparison of GI, PLI and PD at sampling sites between three groups.

Clinical periodontal index	Group	n	T0			T1			T2			T3			*P*					
			P_25_	P_50_	P_75_	P_25_	P_50_	P_75_	P_25_	P_50_	P_75_	P_25_	P_50_	P_75_	T0–T1	T0–T2	T0–T3	T1–T2	T1–T3	T2–T3
PLI	VFR	16	3.00	3.00	4.00	1.25	2.00	2.00	1.25	2.00	2.00	3.00	2.00	3.00	0.01*	0.01*	0.01*	0.65	0.07	0.02*
	HR	16	3.00	3.00	4.00	1.25	2.00	2.00	2.00	2.00	2.00	1.00	1.00	2.00	0.01*	0.01*	0.01*	0.94	0.68	0.58
	LR	16	3.00	3.00	4.00	1.25	2.00	2.00	1.25	2.00	2.75	1.00	2.00	2.00	0.01*	0.02*	0.01*	1.00	0.97	0.37
	*P* value		0.87			1.00			0.99			0.01#								
GI	VFR	16	2.00	2.00	2.00	1.00	1.00	2.00	1.00	1.00	2.00	1.00	2.00	2.00	0.01*	0.01*	0.01*	0.68	0.03	0.01*
	HR	16	2.00	2.00	2.00	1.00	1.00	1.00	1.00	1.00	2.00	1.00	1.00	2.00	0.01*	0.01*	0.01*	0.54	0.69	0.63
	LR	16	2.00	2.00	2.00	1.00	1.00	2.00	1.00	1.00	2.00	1.00	1.33	2.00	0.01*	0.02*	0.01*	0.47	0.35	0.57
	*P* value		0.39			0.78			1.00			0.42								
PD	VFR	16	2.33	2.33	2.67	1.00	1.33	1.33	1.33	1.33	2.00	2.00	2.00	2.00	0.02*	0.01*	0.01*	0.65	0.04*	0.02 *
	HR	16	2.33	2.33	2.67	1.33	1.33	1.88	1.33	1.33	1.50	1.33	1.33	1.33	0.02*	0.01*	0.01*	0.57	1.00	0.63
	LR	16	2.33	2.33	2.67	1.08	1.33	2.00	1.00	1.33	2.00	1.33	1.33	1.88	0.01*	0.01*	0.01*	0.74	0.85	0.68
	*P* value		0.34			0.79			0.96			0.03#								

VFR, vacuum-formed retainer; HR, Hawley retainer; LR, Lingual retainer.

*Bonferroni multiple comparison test result (intragroup comparisons), Significant difference.

^#^Bonferroni multiple comparison test result (intragroup comparisons), Significant difference.

The correlation analysis between the changes of Pg and Aa counts and PD changes showed that that there was no significant correlation between Pg and PD, or between Aa and PD. There was a positive correlation between Pg and Aa levels (r = 0.529; *p* < 0.033).

## Discussion

The purpose of this study was to investigate the effects of three different orthodontic retainers on changes in Pg and Aa content and periodontal parameters in patients within 6 months of use. Numerous studies have investigated changes in periodontal parameters and microorganisms during orthodontic treatment with fixed orthodontics^4, 18^, but only a few studies have investigated the changes in periodontal microflora and periodontal parameters during the use of orthodontic retainers^19^. In clinical practice, three orthodontic retainers are commonly used: VFRs, HRs, and LRs. The choice of retainer is influenced by several factors, including periodontal health, esthetics, retainer effects, and cost. The size and composition of materials differ among retainers. The VFR is a vacuum-formed hot plastic retainer made of polyvinylchloride (PVC) that covers the entire dental arch^20^. The HR contains a metal snap ring and an acrylic splint that covers part of the dental arch. In contrast, the LR is made of twisted metal wires bonded to six front teeth using resin^21^. These retainers can cause plaque accumulation, reduce cleaning effectiveness, and affect periodontal status. Accordingly, it is necessary to compare the effects of the three retainers on the oral flora and periodontal parameters.

There were no significant differences in periodontal parameters and bacterial content among the three groups of retainers at T0. T0 represents baseline data prior to un-bonding of the bracket, which may simulate the bacterial and periodontal conditions during orthodontic treatment. We observed that oral hygiene and periodontal status significantly improved 1 month after bracket removal. This is because the presence of metal brackets affects oral hygiene and increases retention of dental plaques. The removal of metal brackets permits more effective removal of dental plaques, improvements in periodontal condition, and reduction of plaque adhesion.

Studies on Pg and Aa have reported that 1 month after bracket removal, the content of both bacteria was significantly decreased regardless of the type of retainer used. These findings are consistent with the results of Sallum et al.^22^, which demonstrated that the content of *Porphyromonas gingivalis* and *Aggregatibacter actinomycetemcomitans, Bacteroides forsythus,* and *Prevotella intermedia* was significantly decreased 1 month after removal of the orthodontic appliance. In this study, we followed up patients at 3 and 6 months, and observed that regardless of the type of retainer, Pg and Aa levels remained significantly lower than baseline levels. As Kinane et al.^23^ have reported, the build-up of these plaques is responsible for gingivitis. Lower levels of bacteria in the saliva are indicative of improved oral hygiene. In a study by Jung et al.^24^, 43 of the 58 participants received removable and lingual retainers concurrently, consistent with our findings, the authors reported a significant decrease in total bacterial levels in the saliva at 5 weeks, alongside improved oral hygiene.

At the first third month, there was no significant difference in the content of Pg and Aa between the three groups (*P* > 0.05), but at the sixth month, there was a difference. At T3, Aa content was the lowest in the HR group and was significantly lower in the HR group than in the VFR and LR groups (*p* < 0.05), while there was no significant difference between the VFR and LR groups (*p* > 0.05). This suggests that HR may have a lesser impact on periodontal disease. At T3, Pg content was the lowest in the HR group, and the difference was statistically significant (*P* < 0.05). There was no significant difference between the VFR and LR groups (*P* > 0.05). Rody et al.^25^ investigated the changes in biomarker levels in gingival crevicular fluid of patients wearing different orthodontic retainers, and observed that biomarker levels were lower in the HR group, suggesting that the HR had less of an impact on periodontal health. Notably, this study demonstrated that there was a tendency for bacterial levels to increase in the VFR group at T3. This may suggest that long-term use of VFR increases periodontal risk.

Aa is a gram-negative anaerobe that can survive in the oral cavity even after the removal of metal appliances due to its facultative anaerobic characteristics. Aa helps obligate anaerobes such as *Porphyromonas gingivalis* and *Tannerella forsythia* to survive in the oral cavity^26^. In accordance with this, PCR detection of Pg and Aa in saliva still revealed a certain level of bacteria even after 6 months. This suggests that the use of retainers is not the cause of the presence of Pg and Aa in the mouth. However, after removing the metal bracket, the content of Pg and Aa decreased, and the content of Aa in the mouth was higher than that of Pg.

Our analysis of periodontal parameters revealed that the PLI, GI, and PD of the three groups were significantly lower after the removal of metal brackets. This means that the periodontal tissue is gradually recovering after 1 month of bracket removal. In the first month, there were no significant differences in PLI, GI, and PD among the VFR, HR, and LR groups, with all groups showing improvements in oral condition. However, at the sixth month, significant differences in periodontal parameters were observed between the groups, whereby PLI and PD were the highest in the VFR group and the lowest in the HR group. Although the periodontal parameters of the VFR group did not reach T0 levels at this time, they were significantly higher than those at T1 and T2. Thus, long-term use of VFR contributed to increased dental plaque formation. Although PLI, GI, and PD exhibited an upward trend in the LR group at the sixth month, no significant difference was observed which indicates that the use of a lingual retainer does not cause periodontal tissue damage after six months. As such, compared with HR and LR, VFR had a greater impact on periodontal health after 6 months of use, and HR had the least impact. In addition, a significant improvement in periodontal status was observed after removal of the orthodontic appliance relative to baseline. Liu et al. and Kim et al.^19, 27^ reported that periodontal conditions improved significantly after the removal of orthodontic appliances, and were significantly lower than baseline. Previous Meta analyses^28^ have demonstrated that compared to VFR, HR have better effects on periodontal health, especially PLI after 6 months. Interestingly Storey et al.^6^ reported that periodontal parameters in the VFR group were superior to those in the LR group, whereas our study showed that the LR group was superior to the VFR group. This inconsistency could be caused by differences in shape of the LR. In this experiment, we chose a nickel-titanium flat wire, which was small in size and the material’s texture was not conducive to plaque accumulation.

As HR does not cover the tooth surface, saliva can be used for self-cleaning to maintain oral hygiene. Conversely, VFR cover all the tooth surfaces and as the transparent film retainer is wound on all tooth surfaces for a long time, the self-cleaning effect of saliva on the tooth surface is seriously affected. The residue and soft scale attached to the retainer surface, and the socket and groove of the retainer are not easy to clean, leading to plaque accumulation and irritation. Moreover, VFR is mainly composed of polyvinyl chloride, and wear and scratches can easily increase its surface roughness, which is conducive to the attachment of bacteria, and the formation of biofilms. Furthermore, its uneven edges may be a stimulus to the periodontal tissue^29^. In the process of the experiment, we also found that even if the patients cleaned the retainer in their daily life, there were still different degrees of soft scale attached to the surface of the retainer, especially on the inner surface of the transparent pressure film retainer. These factors may have contributed to the increase in clinical indexes after 6 months of use. Clinically, it can also be seen that after patients have worn VFR for a long time, its surface has an occasionally odorous, white soft scale attachment. Although bacterial levels did not reach baseline at T3, this suggests that prolonged wearing of VFR without proper cleaning may pose a risk to periodontal health. At the T3 stage, the bacterial Aa content was the least in the HR group, which suggested that the HR group had less periodontal damage than the other two groups. In this study, a nickel-titanium flat wire was used for LR, which demonstrated a better fit on the lingual surface of the six front teeth and was perceived as less of a foreign body. Compared with the traditional round twist wire, LR was more convenient for oral cleaning and was therefore in a relatively stable state during follow-up.

The depth of periodontal probing is an important indicator of periodontal health. Some studies believed that the depth of periodontal probing is correlated with subgingival microbiota. In this study, the correlation analysis between the changes of Pg and Aa content and the depth of periodontal probing in each retainer group showed that there was no correlation between the depth of periodontal probing and the change of single bacterial content in either group. Further studies are needed to determine whether the depth of periodontal probing is correlated with the changes in the content of certain bacteria.

This study has several limitations. We selected only four index teeth for periodontal measurement, which may not be fully representative of all teeth. Furthermore, orthodontic retainers generally need to be worn for more than 2 years, and a follow-up of 6 months is insufficient. As such, further long-term research is needed to investigate the effect of the retainer on periodontal health.

## Conclusions

The following conclusions can be drawn from this study. Firstly, after the removal of metal brackets, the periodontal tissue condition improved regardless of which orthodontic retainer was used, and the contents of Pg and Aa in the oral cavity decreased significantly. Secondly, with regard to Pg and Aa content and periodontal clinical parameters, the HR was superior to the VFR and the LR after 6 months of wear. Thirdly, there was no significant correlation between the contents of *Porphyromonas gingivalis* and *Aggregatibacter actinomycetemcomitans* and periodontal probing depth.

### Supplementary Information

Supplementary Information 1.

Supplementary Information 2.

Supplementary Information 3.

Supplementary Information 4.

Supplementary Information 5.

Supplementary Information 6.

Supplementary Information 7.

## Data Availability

All data generated or analyzed in this study are included in this published article (and its supplementary information file).
