# Supplementary material for: Effects of three orthodontic retainers on periodontal pathogens and periodontal parameters
Source: Sci Rep. 2023 Nov 24;13:20709. doi: 10.1038/s41598-023-46922-2 (PMC10673872; doi:10.1038/s41598-023-46922-2)
Supplement: Supplementary file 3 — Supplementary Information 3. [file 41598_2023_46922_MOESM3_ESM.doc]

Supplemental Table :

Scoring system for the Gingival index

| Score | Criteria |
| --- | --- |
| 0 | Gingiva pink-pale pink. Surface is matt after drying. Degree of stippling varies. Gingival margin may be located on the enamel or at various levels apical to the cementoenamel junction. Tip of the papilla should be the most incisally/occlusally located part of the gingiva. On palpation with a blunt instrument the gingiva should be firm. |
| 1 | Mild inflammation. Gingival margin slightly more red/blue-red than normal and there is a slight oedema of the margin. A colourless gingival exudate may be observed. Bleeding not provoked when a blunt probe is run along the soft tissue wall of the entrance of the gingival crevice. |
| 2 | Moderately inflamed gingiva. Gingiva red/red-blue and glazy. Enlargement due to oedema. Bleeding provoked when a blunt probe is run along the soft tissue wall of the entrance of the gingival crevice. |
| 3 | Severe inflammation. Gingiva markedly red/red-blue and enlarged. Tendency to spontaneous bleeding. Ulceration. |

Scoring system for the Plaque Index.

| Score | Criteria |
| --- | --- |
| 0 | Smear the plaque on the tooth surface with plaque display agent, gargle and check the distribution range of colored plaque on the tooth surface, the tooth surface without plaque |
| 1 | There are scattered spotty plaque at the gingival margin of the neck of teeth |
| 2 | The continuous narrow band plaque width of dental neck should not exceed 1mm |
| 3 | The area of plaque covering the neck of teeth was more than 1mm, but less than 1/3 of the surface of teeth |
| 4 | Plaque covers at least 1/3 of the dental surface, but not more than 2/3 |
| 5 | The area covered by plaque is 2/3 or more than 2/3 of the tooth surface |
