# Supplementary figures and images for: Effects of three orthodontic retainers on periodontal pathogens and periodontal parameters
Source: Sci Rep. 2023 Nov 24;13:20709. doi: 10.1038/s41598-023-46922-2 (PMC10673872; doi:10.1038/s41598-023-46922-2)

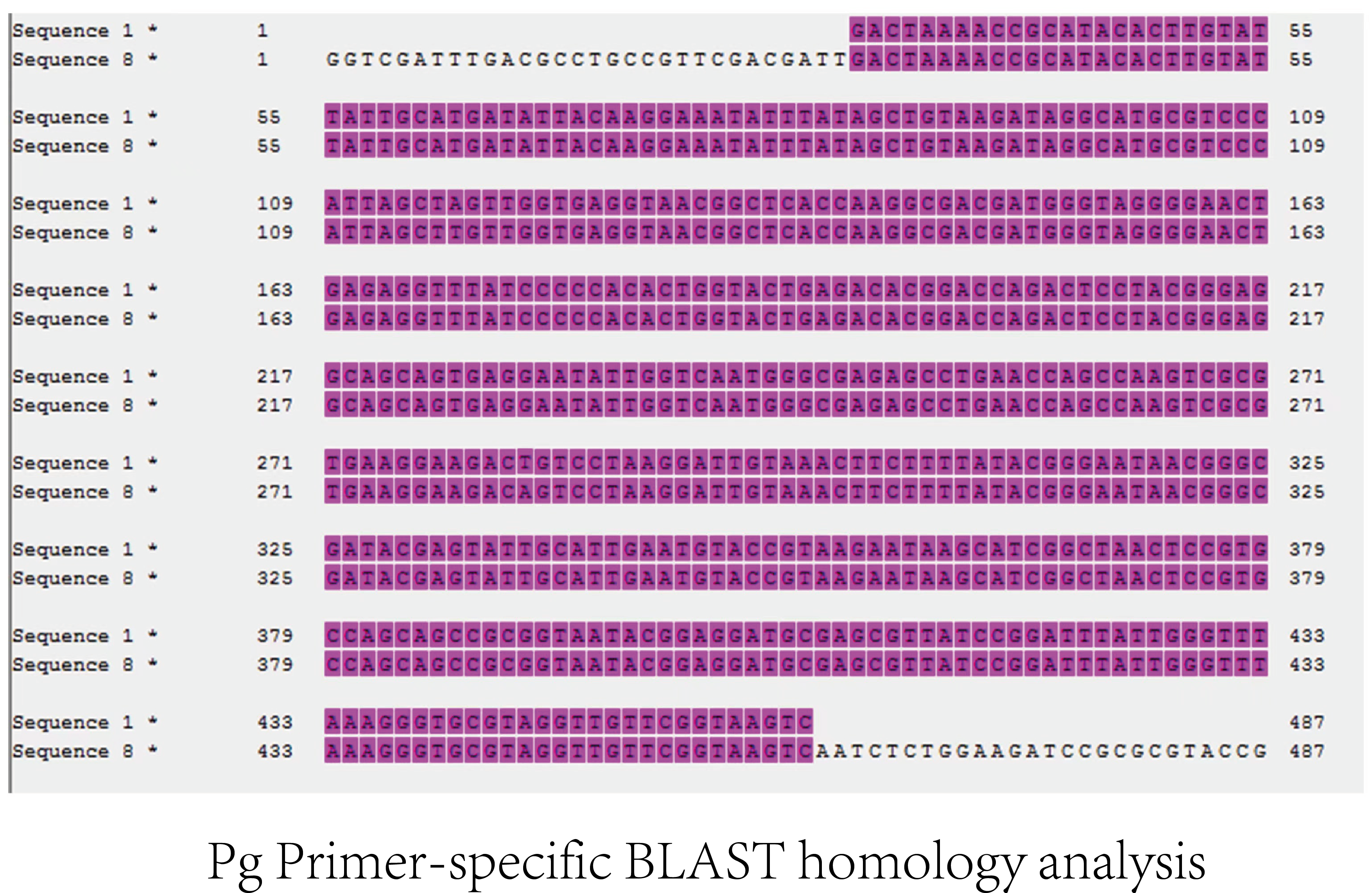

Supplement: Supplementary file 4 — Supplementary Information 4. [file 41598_2023_46922_MOESM4_ESM.png]

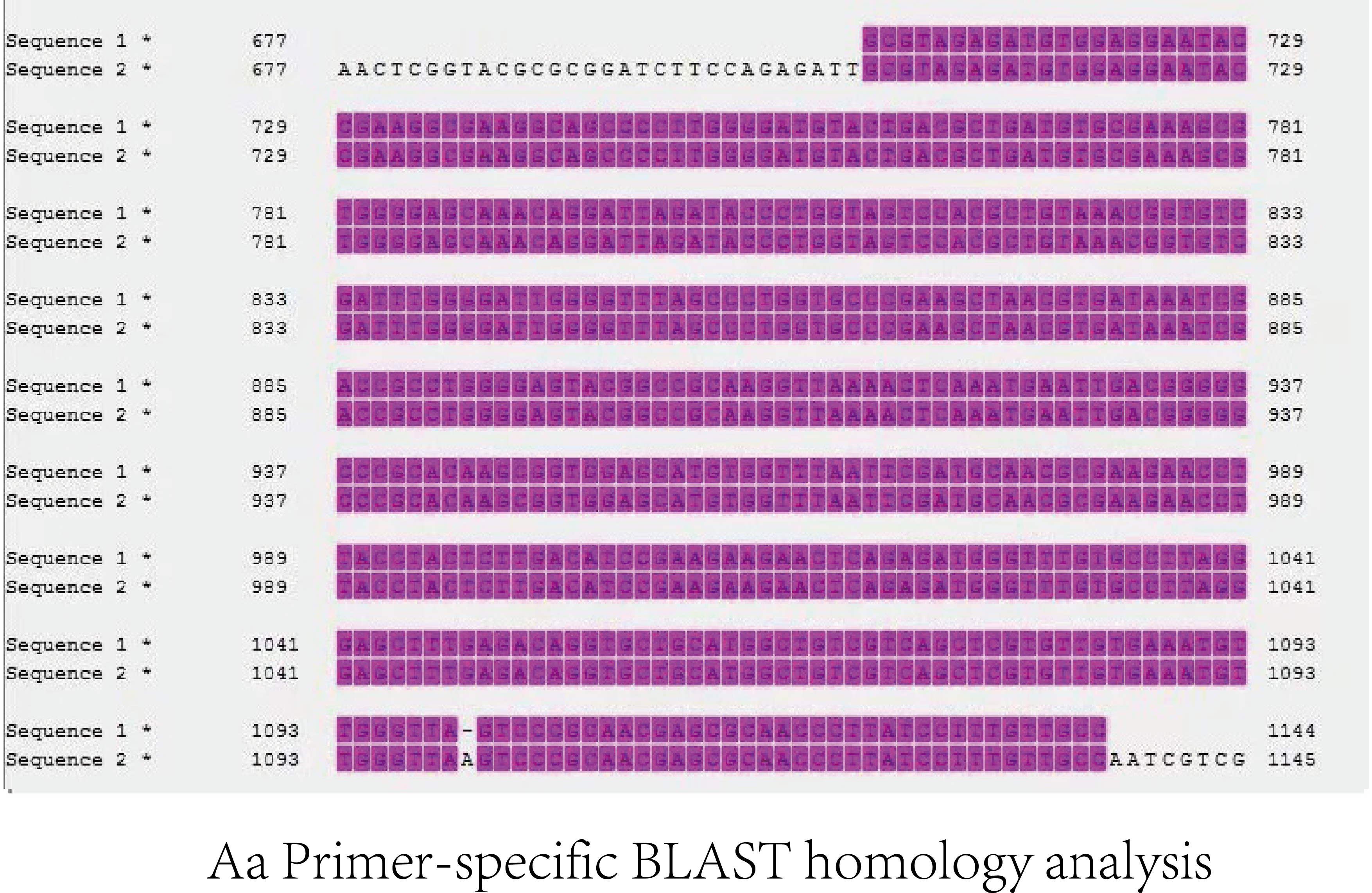

Supplement: Supplementary file 5 — Supplementary Information 5. [file 41598_2023_46922_MOESM5_ESM.png]
