# Supplementary material for: Effects of three orthodontic retainers on periodontal pathogens and periodontal parameters
Source: Sci Rep. 2023 Nov 24;13:20709. doi: 10.1038/s41598-023-46922-2 (PMC10673872; doi:10.1038/s41598-023-46922-2)
Supplement: Supplementary file 6 — Supplementary Information 6. [file 41598_2023_46922_MOESM6_ESM.docx]

The reaction system:

| Reagent | Volume（μl） |
| --- | --- |
| M13 F（2μM） | 2.5 |
| M13 R（2μM） | 2.5 |
| dNTP(2.5mM) | 2 |
| Taq DNA polymerase | 0.5 |
| 10×Taq DNA polymerase buffer | 2.5 |
| bacteria solution | 1 |
| ddH_2_O | Up to 20 |

The reaction conditions：

94℃10min；94℃ 30 sec，50℃ 30 sec，72℃ 30 sec, 30 cycles；72℃ 10 min，4℃ 5 min。
