# Supplementary material for: Effects of three orthodontic retainers on periodontal pathogens and periodontal parameters
Source: Sci Rep. 2023 Nov 24;13:20709. doi: 10.1038/s41598-023-46922-2 (PMC10673872; doi:10.1038/s41598-023-46922-2)
Supplement: Supplementary file 7 — Supplementary Information 7. [file 41598_2023_46922_MOESM7_ESM.docx]

**Research Protocol**

Project leader: Zeng Xiantao (Shanxi Medical University)

Department: Dental multidisciplinary Diagnosis and Treatment Center

Contact person: Li Bowen

Contact number: 18835570012

Project source: None

The study period was from August 2019 to June 2022

Version number: V1.0

Version date: 27 August 2019

**Effects of three orthodontic retainers on periodontal pathogens and periodontal parameters**

Bowen Li^1^, Cailian Lu^2^, Xinhui Yao^1^, Xiaojun Wu^1^, Guilin Wu^1^, Xiantao Zeng ^1^*

*Correspondence:zengxiantao520521@163.com

1. Department of Stomotology, Longgang Otolaryngology hospital,Shenzhen 517172, People’s Republic of China
2. Department of Stomatology, The First Hospital of Shanxi Medical University, Taiyuan 030001, People’s Republic of China.

**Introduction**

After orthodontic treatment, periodontal tissue and alveolar bone remodeling occurs more slowly than changes in the teeth, and are affected by occlusal interferences and growth factors, the tooth will bounce back to its starting position^[1]^. Studies have shown that about 70 to 80 percent of orthodontic patients have a relapse for a variety of reasons^[2]^. Therefore, the use of orthodontic retainers is a critical component of orthodontic treatment and various orthodontic retainers are currently available for clinical application. However, the retainer is a foreign body in the oral environment, which may disturb the dynamic balance of oral microbes, and consequently affect periodontal health.

In the process of orthodontic treatment, the use of fixed orthodontic appliances may hinder cleaning measures and self-cleaning oral hygiene practice, resulting in increased risk of plaques, changes in oral microflora, deterioration of oral hygiene, and gingival inflammation^[3,4]^. In the maintenance phase of orthodontic treatment，several issues remain of concern to both doctors and patients, including whether the use of various retainers can restore periodontal tissue health, the duration of restoration, the choice of retainer with the least impact on periodontal tissue, and the risk of damage to periodontal tissue. Currently, vacuum-formed retainers (VFR), Hawley retainers (HR), and lingual fixed retainers (LR) are commonly used in clinical practice.

Studies have reported that orthodontic treatment may cause an imbalance in the local oral microecology, resulting in changes in the oral flora of patients^[5,6]^. A proportion of conditioned pathogenic bacteria may become dominant and contribute to periodontal tissue changes, such as gingivitis and periodontitis. The occurrence and development of periodontitis are related to various subgingival pathogenic bacteria, such as *Porphyromonas gingivalis* (Pg) and *Aggregatibacter actinomycetemcomitans* (Aa), which are well-documented periodontal pathogens^[7]^. Pg is one of the most widely studied and well-documented periodontitis—causing bacteria. Pg has the highest detection rate of subgingival plaques in patients with chronic periodontitis, and has also been detected in healthy individuals^[8]^. Aa is associated with adolescent periodontitis as well as other types of periodontitis, and can also be detected in healthy individuals. Growing evidence suggests that Aa is closely associated with invasive periodontitis^[9]^.

Developments in biotechnology have improved methods for detection of periodontal microorganisms. In particular, real-time PCR, a relatively novel method based on conventional PCR detection, is typically employed to determine the number, and species, of specific bacteria^[10]^. The basic principle of this approach involves the addition of fluorescent substances that specifically mark PCR products into the PCR reaction, and harnessing the accumulation of fluorescence signals to monitor the entire PCR process in real time. This process produces an S-type amplification curve. If the initial PCR curve conforms to exponential amplification, the molecular number of the initial template can be determined indirectly by comparing the cumulative time of products based on a simple exponential equation, and the template can be quantitatively analyzed using a standard curve^[11,12]^.

**Objective**

Recent studies have reported retention effect, survival, and patient satisfaction with different retainers^[6,13,14]^. However, there is a paucity of studies on periodontal tissue recovery after removal of fixed appliances, and quantitative changes in periodontal pathogens during appliance wear. Further, in a 2016 Cochrane review of orthodontic retainers, the authors emphasized the need for more randomized controlled studies to compare the effects of different retainers on periodontal conditions^[15]^. The changes of Pg and Aa levels in the oral cavity during the retention stage after orthodontic treatment were analyzed by real-time fluorescence quantitative PCR. The effects of VFR, HR and BR on periodontal pathogens and periodontal status were compared.

**Trial Methods**

The study procedures were approved by the Medical Ethics Committee of the first Hospital of Shanxi Medical University in November 2019 (No.2019k033). All patients in the study signed informed consent forms.Participants were recruited to the study from November 2019 through November 2021. The study population consisted of healthy patients who had successfully completed orthodontic treatment with fixed orthodontics and were scheduled to enter the maintenance phase. The inclusion criteria were as follows: (1) both upper and lower dental arches were treated with fixed orthodontic treatment, and patients were satisfied with the orthodontic results, (2) patients used brackets with a 0.022-inch groove (3M Unitek) and common metal brackets, (3) more than 24 permanent teeth, (4) patients could provide good oral health care, and (5) correction period of > 12 months. The exclusion criteria were as follows: (1) systemic diseases, (2) use of any drugs that may have affected periodontal health (such as antibiotics) during the study period, (3) severe or uncontrolled caries, (4) cleft palate or severe facial deformities, (5) any active periodontal disease, (6) dentures in the mouth, (7) smokers, and (8) pregnant patients.

The size of the study population was predetermined using power analysis in R version 4.1.1. Based on a 1:1 ratio between groups, a total sample size of 45 patients would provide a power of more than 80%.

VFR group, HR group, and LR group. Participants were randomly assigned using a lottery technique. An equal number of pieces of paper labelled with the names of different retainers were placed into a sealed opaque box. Participants arbitrarily selected which retainer to use via a lottery approach. Due to the nature of the trial, it was not possible to completely blind clinicians and participants.

Patients were assessed at removal of the bracket (T0) and after 1 month (T1), 3 months (T2), and 6 months of wearing the retainer (T3). Evaluation parameters included gingival index (GI), plaque index (PLI), probing depth (PD), and the periodontal pathogens content of Pg and Aa. All assessments and retainers were performed by two orthodontists trained as periodontists.

Index teeth 16, 21, 31, and 46 were selected to measure the periodontal clinical indicators. Patients were required to refrain from eating or brushing their teeth for 2 h before the test. In this study, the GI adopted the scoring standard proposed by Loe and Silness^[16]^, and the PLI adopted the scoring standard proposed by Quigley and Hein^[17]^. A detailed scoring standard is presented in S1 Appendix. PD was determined using the periodontal probe KPC15 (Kangqiao; Shanghai, China). The distance from the bottom of the periodontal pocket, or gingival trench, to the gum margin was measured. Six sites were detected for each index tooth, with three measurements per site, and the average value was obtained. At the time of T0 sampling, the patient had not removed the metal bracket. The data collected at T0 therefore represented the patient's periodontal condition and bacterial status during orthodontics, and this timepoint was considered the baseline.


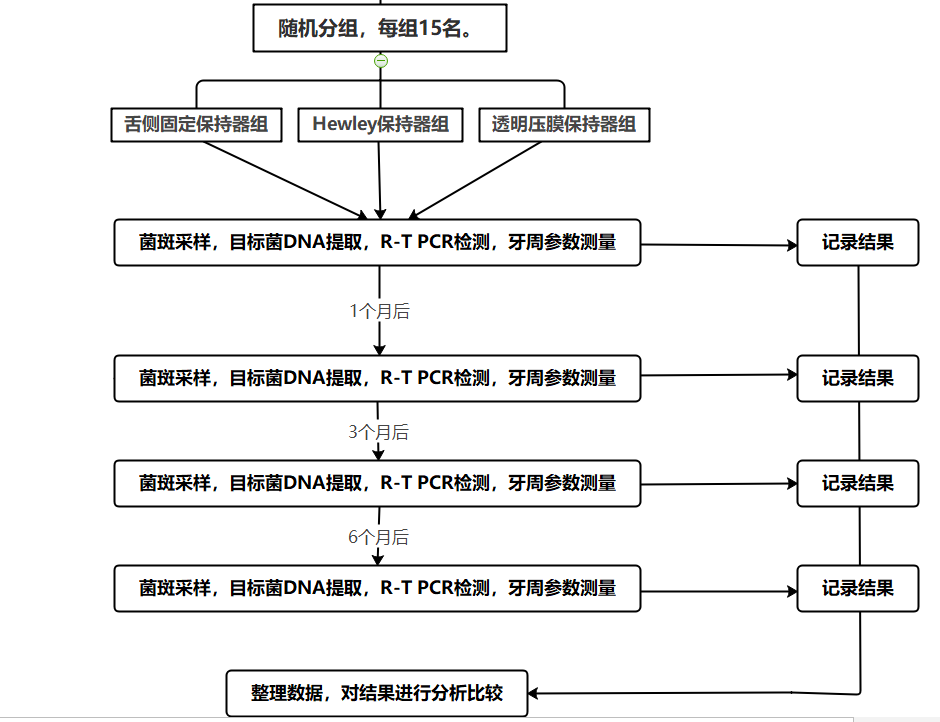


**Flow chart**

**Criteria for evaluating efficacy**

The content of porphyromonas gingivalis was changed, and the content of actinobacillus was changed. PLI GI PD.

**Observation of adverse events**

The orthodontic retainers used in this study were for routine clinical use and were not involved in adverse events.

**Ethics of clinical research**

The clinical study will follow the World Medical Assembly Declaration of Helsinki and other relevant regulations. The clinical study was carried out after approval of the study protocol by the ethics committee prior to the initiation of the study. Before each subject is enrolled in this study, the investigator is responsible to provide the subject or his/her surrogate with a complete and comprehensive introduction of the purpose, procedures and possible risks of this study, and sign a written informed consent form. Subjects should be informed that they have the right to withdraw from this study at any time, and the informed consent should be kept as a clinical research document for future reference. The personal privacy and data confidentiality of the subjects will be protected during the study.

**Statistical analysis**

The SPSS software package was used for data analysis (Version 20; SPSS, USA), with statistical significance set at 5%. Data were assessed for normality using the Shapiro–Wilk normality test. The Levene test was used to assess homogeneity of variance. The results of the normality test indicated that the data of periodontal parameters did not conform to a normal distribution, while the Pg and Aa data were normally distributed with a uniform variance. Therefore, the Kruskal−Wallis test was used for overall inter-group comparisons of periodontal parameters, whereas the Friedman test and Nemenyi post-test were used for intra-group comparisons, the data in the tables are described in quartiles. Univariate ANOVA was used for overall comparison of bacteria between groups, and Bonferroni-corrected test results were used for intra-group comparisons, the data in the table are described as mean ± standard deviation. Log 10 transformation was performed on the microbiologic data so that the distribution was normalized and the variance was stabilized. Spearman’s correlation coefficient was used to analyze the correlation between the periodontal clinical parameters, Pg, and Aa.

**Progress of the study**

From November 2019 to November 2021

The training and consistency assessment of outpatient case collection personnel were completed. The saliva collection and clinical measurement of the enrolled samples of the study group were completed according to the study protocol. From September 2021 to October 2021 。The collected samples were detected, analyzed, and the experimental indexes were measured. From November 2021 to June 2022 Start the summary work of the project and complete the academic and scientific research activities related to the project conclusion.

Complete the arrangement and summary of the project data, summarize the experience, etc

**Participants**

Name Title/Professional assignment GCP/ Ethics Training (time)

Prof. Xiantao Zeng/Orthodontics Project Design 2020.12

Dr. Lu Cai-lian/Orthodontic Patient Enrollment and Measurement data 2020.12

Dr. Li Bo-wen/Statistical Analysis of Orthodontics Data 2020.12

**References**

[1] Melrose C, Millett D T. Toward a perspective on orthodontic retention?[J]. Am J Orthod Dentofacial Orthop, 1998, 113(5): 507-14.

[2] Littlewood S J, Kandasamy S, Huang G. Retention and relapse in clinical practice[J]. Aust Dent J, 2017, 62 Suppl 1: 51-57.

[3] Klukowska M, Bader A, Erbe C, et al. Plaque levels of patients with fixed orthodontic appliances measured by digital plaque image analysis[J]. Am J Orthod Dentofacial Orthop, 2011, 139(5): e463-70.

[4] Baka Z M, Basciftci F A, Arslan U. Effects of 2 bracket and ligation types on plaque retention: a quantitative microbiologic analysis with real-time polymerase chain reaction[J]. Am J Orthod Dentofacial Orthop, 2013, 144(2): 260-7.

[5] Ristic M, Vlahovic Svabic M, Sasic M, et al. Clinical and microbiological effects of fixed orthodontic appliances on periodontal tissues in adolescents[J]. Orthod Craniofac Res, 2007, 10(4): 187-95.

[6] Moslemzadeh S H, Sohrabi A, Rafighi A, et al. Comparison of Stability of the Results of Orthodontic Treatment and Gingival Health between Hawley and Vacuum-formed Retainers[J]. J Contemp Dent Pract, 2018, 19(4): 443-449.

[7] Heaton B, Dietrich T. Causal theory and the etiology of periodontal diseases[J]. Periodontol 2000, 2012, 58(1): 26-36.

[8] Paster B J, Boches S K, Galvin J L, et al. Bacterial diversity in human subgingival plaque[J]. J Bacteriol, 2001, 183(12): 3770-83.

[9] Haubek D, Ennibi O K, Poulsen K, et al. The highly leukotoxic JP2 clone of Actinobacillus actinomycetemcomitans and progression of periodontal attachment loss[J]. J Dent Res, 2004, 83(10): 767-70.

[10] Nonnenmacher C, Dalpke A, Rochon J, et al. Real-time polymerase chain reaction for detection and quantification of bacteria in periodontal patients[J]. J Periodontol, 2005, 76(9): 1542-9.

[11] Braga R R, Carvalho M A, Bruña-Romero O, et al. Quantification of five putative periodontal pathogens in female patients with and without chronic periodontitis by real-time polymerase chain reaction[J]. Anaerobe, 2010, 16(3): 234-9.

[12] Hyvärinen K, Laitinen S, Paju S, et al. Detection and quantification of five major periodontal pathogens by single copy gene-based real-time PCR[J]. Innate Immun, 2009, 15(4): 195-204.

[13] Forde K, Storey M, Littlewood S J, et al. Bonded versus vacuum-formed retainers: a randomized controlled trial. Part 1: stability, retainer survival, and patient satisfaction outcomes after 12 months[J]. Eur J Orthod, 2018, 40(4): 387-398.

[14] Jin C, Bennani F, Gray A, et al. Survival analysis of orthodontic retainers[J]. Eur J Orthod, 2018, 40(5): 531-536.

[15] Littlewood S J, Millett D T, Doubleday B, et al. Retention procedures for stabilising tooth position after treatment with orthodontic braces[J]. Cochrane Database Syst Rev, 2016, 2016(1): Cd002283.
